# Polysaccharide-Rich Fractions from *Rosa rugosa* Thunb.—Composition and Chemopreventive Potential

**DOI:** 10.3390/molecules24071354

**Published:** 2019-04-06

**Authors:** Marta Olech, Natalia Nowacka-Jechalke, Maciej Masłyk, Aleksandra Martyna, Wioleta Pietrzak, Konrad Kubiński, Daniel Załuski, Renata Nowak

**Affiliations:** 1 Chair and Department of Pharmaceutical Botany, Medical University of Lublin, 1 Chodźki Street, 20-093 Lublin, Poland; natalianowacka@umlub.pl (N.N.-J.); wioleta.pietrzak@umlub.pl (W.P.); renatanowak@umlub.pl (R.N.); 2 Department of Molecular Biology, Faculty of Biotechnology and Environmental Sciences, The John Paul II Catholic University of Lublin, Konstantynów 1i Street, 20-708 Lublin, Poland; maciekm@kul.lublin.pl (M.M.); aleksandra.martyna@kul.lublin.pl (A.M.); kubin@kul.lublin.pl (K.K.); 3 Department of Pharmacognosy, Ludwik Rydygier Collegium Medicum, Nicolaus Copernicus University, 9 Marie Curie-Skłodowska Street, 85-094 Bydgoszcz, Poland; daniel_zaluski@onet.eu

**Keywords:** carbohydrates, glucan, polysaccharide-peptide conjugates, anti-inflammatory effect, COX, antiproliferative activity, hyaluronidase inhibition, rugosa rose, Japanese rose

## Abstract

The huge health-beneficial potential of polysaccharides encourages the search for novel sources and applications of these compounds. One poorly explored source of polysaccharides is the rose. The content and biological activity of polysaccharides in rose organs is an almost completely unaddressed topic, therefore, polysaccharide-rich extracts (crude polysaccharides, CPLs) from petals, leaves, hips, and achenes of *Rosa rugosa* Thunb. were studied for their composition and the influence on various cellular processes involved in the development of cancer and other civilization diseases. The study revealed the presence of water-soluble and -insoluble polysaccharides (including β-glucans) and protein-polysaccharide conjugates in rose organs. Rose hips were found to be the most abundant source of polysaccharides. Different polysaccharide-rich extracts showed the ability to inhibit pro-inflammatory enzymes (COX-1, COX-2, hyaluronidase), a radical scavenging effect (against DPPH^•^ and ABTS^•+^), and antiproliferative activity (in the A549 lung and SW480 colon cancer cell lines) in in vitro assays. Therefore, rose crude polysaccharides are very promising and can potentially be used as natural chemopreventive agents.

## 1. Introduction

Polysaccharides constitute a structurally diverse group of macromolecules consisting of repeating monosaccharide residues linked together by glycosidic linkages forming linear structures or containing branched side chains. Due to the huge variability of their structures, they possess a very interesting capacity for carrying biological information. In comparison with proteins or nucleic acids, polysaccharides have been undervalued for a long time, however, they are gaining growing interest among the scientific community nowadays. This applies in particular to polysaccharides from natural sources like plants, fungi, or algae [1,2]. These macromolecules play a key role in different biological processes including cell-cell communication, inflammation, pathogenesis prevention, metastasis, immune responses, or fertilization [3]. Based on the evidence from recent decades, polysaccharides have been found to exhibit antioxidant, antibacterial, antiviral, anti-inflammatory, immunomodulatory and, most importantly, anticancer potential [4,5,6]. Different mechanisms of action of polysaccharides in cancer treatment have been recognized. They were found to elicit both direct anticancer activity through induction of cancer cell apoptosis and indirect activity by stimulation of the immune system. Some polysaccharides are already known to exert an immunopotentiation effect in combination with chemotherapy [7]. Moreover, these sugars show anti-inflammatory properties occurring through the ability to down-regulate pro-inflammatory enzymes like cyclooxygenase (COX) isoforms, especially COX-2 [8]. Another enzyme related to the inflammation process is hyaluronidase, which hydrolyses hyaluronic acid, ensuring intracellular viscosity. Fragmentation of hyaluronic acid leads to degradation of the extracellular matrix, which promotes inflammation. Hyaluronidase has also been found to be implicated in tumour invasiveness and metastasis [9]. Since the incidence of cancer and other diseases related to oxidative stress and inflammation is still growing worldwide [10,11], novel strategies to fight against these pathologies are intensively desired. In this context, polysaccharides and polysaccharide-rich extracts provide a promising alternative, and searching for new sources of bioactive natural polysaccharides among plants is reasonable.

*Rosa rugosa* Thunb. possesses a long tradition of being used for food and medicinal purposes in various cultures. Different organs of this plant are known to have various activities such as antioxidant, antimicrobial, antiviral, antidiabetic, or antihypertensive effects due to the abundance of secondary metabolites e.g. phenolic compounds, terpenoids, tannins, or essential oils [12,13,14]. Nevertheless, there is still only little information available about the polysaccharide content and activity in *R. rugosa* species. To date, it has been reported that *R. rugosa* flowers contain a polysaccharide-peptide complex with antioxidant properties [15]. Moreover, Wang et al. [16] revealed antitumor potential of bee pollen polysaccharides from *R. rugosa*.

The aim of this study was to evaluate polysaccharide-rich fractions (crude polysaccharides; CPL) from various *R. rugosa* organs, i.e. petals, leaves, hips, and achenes and to characterize their chemical composition including the content of sugars, proteins, and phenolic compounds. The next step consisted in evaluation of the antioxidant, anti-inflammatory, and anticancer activities of the rose fractions in order to determine their chemopreventive potential.

## 2. Results and Discussion

### 2.1. Content and Composition of Rose Polysaccharide-Rich Fractions

There are scarce data about the composition and biological activity of rose polysaccharides. Polysaccharides from fruits, petals, and achenes of only few rose species have been analysed [15,17,18,19]. To our knowledge, no leaf polysaccharides from any rose species have been studied to date. Therefore, we wanted to examine the content and activity of polysaccharide-rich fractions (crude polysaccharides; CPL) obtained from different parts of rugosa rose, one of the most cultivated, widespread, and consumed rose species. 

The preliminary analyses of the rose organs and polysaccharide-rich fractions indicated that the samples were significantly diverse in terms of the yield and composition of CPLs. There were differences in the content and proportions of water-soluble and -insoluble polysaccharides, sugars, content and proportions of glucans, and amounts of polyphenolics (Table 1 and Table 2). 

The yield of CPL varied greatly depending on the plant part (0.81 to 19.10 g per 100 g of dried plant material), with the highest content recorded for the hips (H). The yield for H was more than two times higher than the yield of the CPL extracted from *R. davurica* and *R*. *roxburghii* fruits (8.61% and 6.59% of d.w., respectively) [18,19]. However, it is hard to compare our results, as the authors of the quoted studies do not specify whether they studied hips with or without achenes. The yield of CPL for the petals, in turn, is similar to that reported by Ng et al. [15] for the same plant material (3.49 g/100 g d.w.). Polysaccharides from seeds of other rose species, i.e. *R. mosqueta*, were examined by Dourado et al. [17], but the amount of obtained CPL was not provided. 

The content of water-soluble sugars in the plant material (g/100 g of dry weight) was calculated from the yield of CPL and sugar content (% of CPL) measured for the analysed plant parts. It can be noticed that the hips contain a significant amount of soluble sugars (15.37 g/100 g d.w.). In the case of other organs, water-soluble sugars represent a relatively small part of dry weight (0.21–0.80 g/100 g d.w.).

β-Glucans are a group of polysaccharides with many confirmed biological properties, e.g. immunomodulatory, prebiotic, anti-inflammatory, and anti-cancer effects [20,21]. We have analysed the ratio of α-glucans to β-glucans and revealed their presence in all samples (Table 1). The proportions of α-glucans to β-glucans varied. The highest glucan content was revealed in the hips (H) and petals (Flos) (12.26 and 6.67 g/100 g d.w., respectively). Lower glucan levels were found in the achenes (Fru) and leaves (Lv), 2.21 and 2.85 g/100g d.w., respectively. For a majority of the examined samples, the content of β-glucans (which are regarded as more valuable and potentially more active; 1.52–6.35 g/100g d.w.) was higher than that of α-glucans (0.70–5.92 g/100g d.w.). A significant amount of petal and achene glucans must be part of the water-insoluble fraction, since their amount exceeds the CPL yield and the content of soluble sugars. The hips also contain a large quantity of glucan molecules, which are almost equally divided into polymers with α- or β- linkages. Considering the proportions of glucans found in a given plant part, β-glycosidic bonds are also more common in the leaves and achenes (twice the amount of α-glucans). On the other hand, α-glucans predominate in the flowers (Flos). It is hard to establish in what proportions glucans represent the water-soluble and -insoluble fractions in H and Lv. The glucans in Flos and Fru mainly belong to water-insoluble polymers. 

The presence of water-insoluble glucan-type polysaccharides was previously observed in *Rosa mosqueta* seeds by Dourado et al. [17]. However, the presence and content of glucans in other organs of the rugosa rose has not been reported to date. Since β-glucans are considered major bioactive sugar compounds related to health promoting activity, including anticancer properties, rose organs might be an interesting source of these constituents. 

It can be noticed than the glucan content in the flowers (Flos) and achenes (Fru) was found to be higher than their CPL yield. This indicates that these plant materials contain a proportionally high amount of glucan-type water-insoluble polysaccharides. During determination of glucans, plant material undergoes direct extraction combined with multi-stage hydrolysis (both acidic and enzymatic). All water-soluble and -insoluble β-glucans and α-glucans are hydrolysed to d-glucose, whose content is measured and used for further calculations. The CLP preparation process, in turn, was carried out to obtain water-soluble polysaccharides. Therefore, the yield refers only to the content of water-soluble compounds extracted from the raw material.

The composition of CPLs from the different *R. rugosa* organs was highly differentiated as well (Table 2). The highest sugar concentration was found for CPL from the hips (80.49%). It was higher than those previously reported for polysaccharides from *R. roxburghii* (63.79%) [22]. CPL-H was found to be the most homogeneous fraction. The composition of other CPLs was more complex. The carbohydrate content in CPL-Flos (20.90%) was similar to that reported by Ng et al. [15] for the same plant material (22.37%). The lowest sugar concentration was found in the leaf crude extract. The CPL from the achenes (fruits) was composed of sugars in more than 25%. 

Only a slight response of CPLs to Bradford’s assay was observed, suggesting a marginal amount of protein in the samples (Table 2.). The highest amount was revealed for the achenes, which are a relatively rich source of protein (after complete removal of the oil, the content is nearly 10% d.w.) [17]. In the other samples, the protein content was < 1%, which was lower than that previously reported for rose CPL. Substantially higher content of proteins (28.46%) was reported in a non-purified polysaccharide fraction from rugosa rose petals [15]. This is probably related to the sample preparation process, where no pre-treatment with ethanol and deproteinization was performed. Another study showed that polysaccharides extracted from *R. roxburghii* fruits contained 4.10% (*w*/*w*) of proteins [22]. In the light of such different observations, we decided to confirm the presence of proteins by SDS-PAGE analysis (the results of which are described below). 

The samples were highly diverse in terms of the phenolic content (0.02–0.22 gallic acid equivalents per g of CPL). The highest level of phenolics was shown in the petals, which are rich in polyphenols [14]. The lowest content was determined for achenes. Therefore, the phenolic content found in CPLs seems to be strongly related to the nature of the plant material [14]. We have observed that the commonly used pre-treatment step with ethanol does not allow removing all phenolics from the plant material. Polyphenols may also be partially linked to polysaccharides, as it was shown that hydroxycinnamates can form intra-and/or intermolecular polysaccharide cross-links in monocotyledonous and dicotyledonous plants [23,24]. 

### 2.2. SDS-PAGE Results

**Figure 1:**
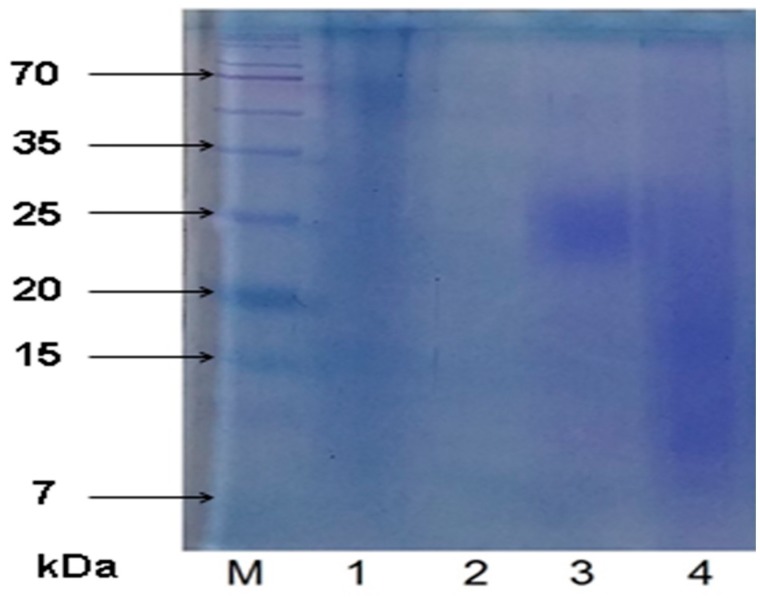
SDS-PAGE analysis of rose CPL. Lanes: M—standard marker; 1—CPL-Flos; 2—CPL-Lv; 3—CPL-H; 4—CPL-Fru.

SDS-PAGE analysis was conducted to confirm the presence of proteins/polysaccharide-peptide conjugates in the CPLs (Figure 1). The presence of the protein component was revealed in the CPL-Flos, CPL-H, and CPL-Fru fractions. The tailing spots on lanes 1, 3, and 4 suggest that these samples contain proteins attached to some other molecules, presumably polysaccharides (the construction of polysaccharides is linear, and they often do not form tight spots) [25]. Previous studies reported a polysaccharopeptide complex in *R. rugosa* flowers [15]. Wang et al. observed proteins in the fraction of water-soluble polysaccharides from R. roxburghii fruits [22]. However, they did not verify whether the protein was linked to a sugar or only co-eluted during extraction. Purified polysaccharide extracted from *R. davurica* fruits did not contain any attached protein [18]. 

In turn, no protein can be observed on the lane of CPL-Lv. No protein-PL linkages were demonstrated in the pectic bee pollen polysaccharide from *Rosa rugosa* tested previously [26]. It is worth mentioning that we have also tested dextran standards (25 and 270 kDa) as a control. We have found that pure polysaccharide is not visible on the CBB-stained SDS-PAGE gel.

The spectrophotometric assay revealed marginal content of proteins in the obtained CPLs (Table 2). However, SDS-PAGE has shown that the proteins are actually present in almost all samples. Moreover, the presence of these proteins may be very important, since they are probably linked to polysaccharides affecting their physical and biological properties. 

Some other combinations of gel electrophoresis with staining procedures were previously used for evaluation of the polysaccharide composition [25]. However, to our knowledge, this is the first study employing SDS-PAGE followed by CBB staining for detection of protein-polysaccharide conjugates in plant samples. Our results indicate that this technique facilitates easy and fast analysis of such complexes. Moreover, it may be used to evaluate the effectiveness of deproteinization procedures. In our case, it allowed demonstrating that the treatment with Sevag reagent removed unbound proteins; however, bound proteins remained in the sample. 

### 2.3. Biological Activity of CPLs

#### 2.3.1. Anti-inflammatory Activity

All samples were tested for anti-inflammatory activity. The ability of several extract concentrations (0.25–5 mg/mL) to inhibit COX-1 and COX-2 enzymes was studied. The potential of the samples was compared with acetylsalicylic acid, which is non-selective NSAID able to inhibit both types of the COX enzyme. 

The CPLs from the petals, leaves, and hips appeared to be non-selective cyclooxygenase inhibitors (Table 3). They inhibited both COX isoenzymes in a dose-dependent manner. CPL-Fru, in turn, was found not to be active in all concentrations tested. 

Significant activity of CPL-Flos was observed for both isoenzymes at the lowest concentrations (0.5 mg/mL and 0.25 mg/mL) and it exceeded 80% at the highest dose tested. Similarly, the COX-2 activity was in over 55% inhibited by CPL-Lv at the lowest dose and the maximum effect observed was 81.1%. On the other hand, the activity of leaf CPL towards COX-1 was moderate. Therefore, CPL-Lv was found to be a more selective COX-2 inhibitor. The activity of CPL-H was visible from the lowest doses, but significant enzyme inhibition was noted for concentrations ≥ 1 mg/mL. The hip polysaccharide-rich fraction at 5 mg/mL inhibited 94.34% and 100% of COX-2 and COX-1 activity, respectively. This effect was comparable with the action of a 1 mM solution of acetylsalicylic acid against COX-2 and was significantly higher than that of the standard solution against COX-1. 

The observed activity of CPLs may largely result from the presence of polysaccharides. Purified polysaccharide from *Rosa davurica* fruits was previously found to exert an anti-inflammatory effect through its impact on several mechanisms involved in development of inflammation [18]. Some other plant-derived polysaccharides were also previously proved to have significant anti-inflammatory activity, influencing multiple targets in the inflammatory process, e.g. decreasing the expression of pro-inflammatory cytokines, increasing the level of anti-inflammatory cytokines, inhibiting NO and prostaglandin-E2 production, affecting the migration of leukocytes into inflammatory sites, and suppressing the activity of lipoxygenases and cyclooxygenases [18]. 

#### 2.3.2. Anti-Hyaluronidase Potential

We assessed all samples for their ability to inhibit hyaluronidase activity. The inhibition of an enzyme that destroys hyaluronic acid can have an impact on the functioning of connective tissue, inflammatory process, tumour invasion, and metastasis. It was shown that all polysaccharide-rich fractions strongly affected hyaluronidase activity (Table 4). CPL-Flos and CPL-Lv were found to be the most effective hyaluronidase inhibitors (IC_50_ 0.40–0.45 mg/mL, respectively). Their potential was comparable or even higher than those observed for the standards. More than twice lower activity was shown by CPL-Fru (1.14 mg/mL), which acted similarly to naringenin. CPL-H exerted the weakest impact on the enzyme function. However, its activity was still higher than that of escin. Similar observations were reported in our earlier paper, where teas and tinctures from flowers and leaves had the strongest anti-hyaluronidase properties among the rose organs studied [14]. The present paper provides the first in vitro evidence of the anti-hyaluronidase potential of rose polysaccharide fractions. It demonstrates that easily available rose materials (especially leaves and petals) are a source of potent natural anti-hyaluronidase molecules.

#### 2.3.3. Antiproliferative Activity

**Figure 2:**
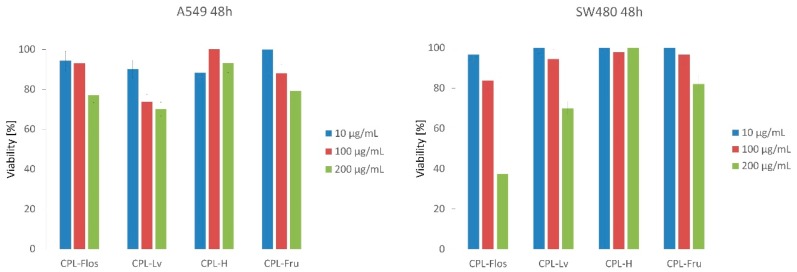
**Figure 2**. Antiproliferative activity of crude rose polysaccharides expressed in % viability. Abbreviations like in Table 2.

A previous study indicated that *R. roxburghii* polysaccharides have potent cytotoxic activity in ovarian cancer A2780 cells and anti-metastatic activity [27]. Therefore, we aimed to examine the cytotoxic potential of our samples. The antiproliferative effect of three concentrations of CPLs was tested on A549 lung cancer and SW480 colon cancer cell lines. In the case of the A549 line, we observed a moderate dose-dependent cytotoxic potential after incubation with the polysaccharide fractions from the petals, leaves, and achenes (true fruits) (Figure 2). These extracts at the concentration of 200 µg/mL decreased the number of living cells by 20.90–29.90%. The strongest effect was observed for CPL-Lv, which killed 26.19% of cancer cells also at the concentration 100 µg/mL. CPL-H demonstrated only slight cytotoxic activity (6.89–11.60% of dead cells). 

The highest cytotoxic effect was observed for CPL-Flos, which significantly reduced the viability of the SW480 cancer cells. At concentrations of 100 and 200 µg/mL, the number of living cells decreased by 22.1% and 62.65%, respectively. The leaf polysaccharide-rich fraction showed considerable antiproliferative activity on the colon cancer line only at the highest concentration studied. In the case of the other CPLs, weak cytotoxic potential towards SW480 was observed. 

Cancer cell lines showed different sensitivity to CPLs-Flos and CPL-Lv. Meanwhile, they were similarly resistant to CPL-H. Differences in cell responses are difficult to interpret at the moment. Further studies are needed to indicate the mechanism of CPLs antiproliferative action. It is worth mentioning that chemoprevention assumes long-term use of food products in order to prevent or inhibit development of cancer. That is why weak or moderate antiproliferative activity is also interesting from the chemopreventive point of view. 

#### 2.3.4. Antioxidant Activity

It is already known and proven that oxidative stress resulting from reaction of powerful radicals with biological molecules induces various diseases and degenerative processes [10]. Among available data, there is voluminous literature regarding the antioxidant activity of plant-derived low molecular weight compounds, while there is insufficient knowledge about the antioxidant activity of polysaccharides. This also refers to *R. rugosa* known from strong antioxidant potential resulting from the presence of e.g. phenolic compounds [28]. 

The present study was undertaken to investigate the antiradical potential of polysaccharide-rich fractions from different parts of *R. rugosa* with the use of two protocols with DPPH^•^ and ABTS^•+^ radicals. The results are presented in Table 5. The highest DPPH^•^ scavenging activity was demonstrated for the polysaccharide fractions from leaves (0.24 mg of dry extract/mg DPPH^•^) and flowers (0.25 mg of dry extract/mg DPPH^•^), while the lowest activity was detected in the rose true fruits (6.88 mg of dry extract/mg DPPH^•^). Similar results were observed in the case of antioxidant activity against the ABTS^•+^ radical cation. Reported values from the TEAC protocol ranged from 0.14 mmol Trolox/g dry extract for true fruits to 1.53 mmol Trolox/g dry extract for flowers. Given the chemical composition of the precipitated polysaccharide-rich fractions, it can be assumed that their antioxidant activity might also result from the presence of phenolic compounds (Table 2). The highest content of phenolics was demonstrated for CPL-Flos, also possessing the highest antiradical activity. However, the *R. rugosa* hips (pseudofruits) revealed interesting antioxidant activity, while the composition of CPL-H showed that sugars constituted a vast majority (80.49%) and phenolic compounds were present only in 10.69%. Therefore, it can be assumed that the radical scavenging potential of CPLs is also associated with polysaccharide activity, bearing in mind that the antioxidant activity of polysaccharides and a polysaccharide-protein complex from *R. rugosa* was demonstrated previously [15,29]. Similarly, Chen and Kan [19] revealed that polysaccharides from *R. roxburghii* fruits had evident DPPH^•^, hydroxyl, and superoxide radical scavenging activities. 

The mechanism of the antioxidant activity of polysaccharides from plants or different sources e.g. algae, fungi, or bacteria is still not completely clear. Current findings indicate that such properties might be related to the chemical structure of polysaccharides. Therefore, investigating the molecular level of antioxidant potential and testing some chemical modifications improving this activity seem to be necessary.

## 3. Materials and Methods

### 3.1. Plant Material and Preparation of Extracts

Petals (Flos), leaves (Lv), hips (H), and achenes (Fru) of *Rosa rugosa* Thunb. were collected in Firlej near Lublin in Eastern Poland in 2017. The petals and leaves were picked at the blossom time. The hips (pseudofruits) with achenes (fruits) were collected in August. The achenes were separated from the red hypanthium. The botanical material was authenticated by Prof. Renata Nowak. A voucher specimen was deposited at the Chair and Department of Pharmaceutical Botany, Medical University of Lublin, Poland (voucher No. R-017/17). The plant material was air-dried at ambient temperature and powdered.

### 3.2. Chemicals

Standards of ascorbic acid, 2,2′-azinobis-(3-ethylbenzothiazoline-6-sulfonic acid) (ABTS^•+^), potassium persulphate, 6-hydroxy-2,5,7,8-tetramethyl-chroman-2-carboxylic acid (Trolox), and 2,2-diphenyl-1-picrylhydrazyl radical (DPPH^•^), thiazolyl blue tetrazolium blue (MTT), bromophenol blue, and Tris–HCl were purchased from Sigma–Aldrich Chemical Co. (St. Louis, MO, USA). β-d-(+)-Glucose was purchased from ChromaDex (Irvine, CA, USA). Dulbecco’s Modified Eagle Medium (DMEM, high glucose + GlutaMAX), fetal bovine serum (FBS), Dulbecco’s phosphate-buffered saline (DPBS) were provided by Gibco (Life Technologies, Paisley, UK). Sodium dodecyl sulphate (SDS) was purchased from BioShop (Burlington, ON, Canada), Coomassie brilliant blue was from Fluka (Buchs, Switzerland), and dithiothreitol (DTT) from Serva (Heidelberg, Germany). Redistilled phenol, sulphuric acid, isoamyl alcohol, and solvents were provided by POCH (Gliwice, Poland). All the chemicals used were of analytical grade.

Assessment of biological activity and determination of total phenolic content was conducted on 96-well transparent microplates (Nunclon, Nunc, Roskilde, Denmark) using an Elisa Reader Infinite Pro 200F (Tecan Group Ltd., Männedorf, Switzerland). Total sugar, glucans, and protein content were determined using a UV-VIS Evolution 300 spectrophotometer (Thermo Scientific, Lafayette, CO, USA).

### 3.3. Extraction of Crude Polysaccharides 

Powdered plant materials were extracted four times with portions of 95% ethanol (1:15 g/mL). They were mixed vigorously twice and macerated at ambient temperature for 48 hours. During the next two extractions, sonication at 35 °C (30 min) was applied. The ethanolic supernatants (containing polyphenols and other small molecular compounds) were removed. Plant residues were air-dried at room temperature and extracted twice with deionized water (1:20 g/mL) with mixing (10 min, room temperature) followed by sonication (1 h, 90 °C) each time. The water extracts were combined and concentrated using a rotary evaporator (at 45 °C) and deproteinized with Sevag reagent (2% isoamyl alcohol in chloroform). After removal of the Sevag reagent, cold anhydrous ethanol (1:4 *v*/*v*) was added to the water phase, and the mixture was kept overnight at 4 °C to precipitate the polysaccharides. The sediment was centrifuged, dissolved in deionized water, and precipitated again with anhydrous ethanol (1:4 *v*/*v*) at 4 °C. The precipitates were then separated by centrifugation at 8000 rpm for 10 min, lyophilized, and weighted to yield the crude polysaccharides (CPL). Three replicate samples were carried out in parallel.

### 3.4. Total Sugar Content

The total sugar content was determined with the phenol-sulphuric acid method [30] using glucose as a standard. 50 µL of an 80% (*w*/*v*) aqueous phenol solution was added to 2 mL of the sample in a test-tube and mixed with 5 mL of concentrated H_2_SO_4_. After 20 minutes at constant temperature of 30 °C in the water bath and cooling for 20 min, the absorbance was measured at 485 nm. The results were converted into mg of glucose equivalents and expressed as % of CPL.

### 3.5. Determination of the Content of Total Glucans as Well as α- and β-Glucans

The content of glucans in the rose organs was determined using an assay kit (Megazyme Ltd., Bray, Ireland) according to the manufacturer’s instructions. The first step involved measuring the content of total glucans including α- and β-glucans plus d-glucose in oligosaccharides, sucrose, and free d-glucose. Milled samples (100 mg) were mixed with 1.5 mL of 10 M HCl. After 45 min of incubation at 30 °C, 10 mL of water were added and the samples were incubated for 2 h at 100 °C. After a neutralization step with 2 M KOH, the samples were adjusted to 100 ml with sodium acetate buffer (pH 5.0). To determine the total glucan content, 0.1 ml of aliquots were mixed with 0.1 mL of exo-1,3-β-glucanase (20 U/mL) plus β-glucosidase (4 U/mL) in 200 mM sodium acetate buffer (pH 5.0) and incubated at 40 °C for 60 min. Then, 3 mL of a mixture consisting of glucose oxidase (>12,000 U/dm^3^) plus peroxidase (>650 U/dm^3^) and 4-aminoantipyrine (0.4 mM) were added to each tube and incubated at 40 °C for 20 min. The absorbances of all solutions were measured at 510 nm against the reagent blank. 

The next steps consisted in measurements of the content of α-glucans plus d-glucose in sucrose and free d-glucose. Milled samples (100 mg) were stirred with 2 mL of 2 M KOH in an ice bath for 20 min. Then, 8 mL of 1.2 M sodium acetate buffer (pH 3.8) were added and the samples were immediately mixed with 0.2 ml of amyloglucosidase (1630 U/mL) plus invertase (500 U/mL) and incubated at 40 °C for 30 min. After enzymatic hydrolysis, 0.1 mL of sodium acetate buffer (200 mM, pH 5.0) plus 3 mL of a mixture consisting of glucose oxidase (>12,000 U/dm^3^) plus peroxidase (>650 U/dm^3^) and 4-aminoantipyrine (0,4 mM) were added to 0.1 mL aliquots and incubated at 40 °C for 20 min. The absorbances of all solutions were measured at 510 nm against the reagent blank including sodium acetate buffer instead of the sample tested. 

The β-glucan content was calculated on the basis of previous results by subtracting the α-glucan content from the total glucan content. All measurements were taken minimum three times. All values of the glucan content were expressed as g/100 g of dry weight and as mean ± standard deviation (SD).

### 3.6. Total Phenolic Content (TPC)

The content of phenolic compounds was assayed using the method described in detail by Olech and Nowak [28]. 

### 3.7. Content of Proteins 

The protein concentration in CPL was determined with the Bradford method as previously described [31].

### 3.8. SDS-PAGE

The samples were mixed with 4X electrophoresis sample buffer (0.125 M Tris–HCl pH 6.8, 4% SDS; 20% *v*/*v* glycerol, 0.2 M DTT, 0.01% bromophenol blue). SDS-PAGE was performed in 10% polyacrylamide gels using a Mini-PROTEAN Tetra Cell device (BioRad, Hercules, CA, USA) according to the Laemmli method [32] at 120 V for stacking gel and 180 V for the resolving gel. The electrophoresis gels were visualized with the use of a staining solution containing Coomassie brilliant blue (CBB).

### 3.9. Anti-Inflammatory Activity (COX)

In vitro anti-inflammatory activity of CPL was determined using the COX (ovine/human) Inhibitor Screening Assay Kit (Cayman, city, MI, USA) following the manufacturer’s instructions. This assay directly measures the amount of prostaglandin 2α (PGF2α) produced by stannous chloride reduction of COX-derived PGH2. Briefly, 10 µL of a polysaccharide sample (0.25 to 5mg/mL, in 5% DMSO) were added to a reaction mixture containing 160μL of assay buffer (0.1 M Tris-HCL, pH-8 with 5 mM EDTA and 2 mM phenol), 10μL of heme, and 10μL of the enzyme (either ovine COX-1 or human recombinant COX-2). After 10-minute incubation at 37 °C, 10 µL of arachidonic acid were added. The reaction mixtures were mixed and incubated for 2 min at 37 °C. Then, 30 µL of a saturated SnCl_2_ solution in 1 M HCl were added to stop COX catalysis. The mixtures were vortexed and incubated for 5 min at room temperature. The prostanoid product was quantified by the enzyme immunosorbent assay (ELISA) using a specific antiserum. The blank contained 10 µl of 5% DMSO instead of the sample and 10 µL of the inactivated enzyme instead of its active form. Aspirin (acetylsalicylic acid, 1mM) was used as a positive control for inhibition of COX-1 and COX-2. The percent COX inhibition was calculated as follows:

COX inhibition activity (%) = (AIAS − ASP)/ AIAS × 100

where ASP = absorbance of the inhibitor sample and AIAS = absorbance of the 100% initial activity sample.

### 3.10. Anti-hyaluronidase Activity

The ability of the extracts to inhibit hyaluronidase was determined using a modified previously described spectrophotometric method [33].

### 3.11. Antiproliferative Activity 

#### 3.11.1. Cell Culture

The A549 (CCL-185) and SW480 (CCL-228) cell lines were obtained from ATCC. The cells were cultured in DMEM + GlutaMAX supplemented with penicillin (50 μg/mL), streptomycin (50 U/mL), and 10% heat-inactivated FBS. They were maintained in a humidified atmosphere at 37 °C and 5% CO_2_ and passaged twice before the experiment.

#### 3.11.2. Antiproliferative Activity Assay 

For the antiproliferative activity assay, the cells of each cell line were seeded in 96-well microplates (Nest Biotechnology, Wuxi, China) at a density of 2.5 × 104 cells/mL in 100 μL DMEM + GlutaMAX supplemented with 10% heat-inactivated FBS. After 24 h of cell attachment, the plates were washed with 100 μL/well of Dulbecco’s phosphate buffered saline (DPBS) and the cells were treated with three concentrations (10 μg, 100 μg, and 200 μg final concentration) of each compound prepared in fresh FBS-free medium for 48h. Each concentration was tested in triplicate. The sets for both cell lines included wells containing sterile water as a negative control. The antiproliferative activity of the compounds was assessed using the MTT assay described below. 

Following 48 h of compound exposure, the control medium or the test exposure medium were removed, the cells were rinsed with DPBS, and 100 μL of fresh medium (without FBS or antibiotics) were added to each well. 10 μL of MTT (5 mg/mL) prepared in DPBS were added to each well and the plates were incubated for 3 h at 37 °C in a 5% CO_2_ humidified incubator. After the incubation period, the medium was discarded, the cells were washed with 100 μL of DPBS, and 100 μL of DMSO were added to each well to extract the dye. The plate was shaken for 10 min and the absorbance was measured at 570 nm. Viability was calculated as the ratio of the mean of OD obtained for each condition to the control condition.

### 3.12. DPPH^•^ Radical Scavenging Activity Assay

The scavenging effect of the samples was tested using a slightly modified method proposed by Olech and Nowak [28]. Aliquots of 180 μL of a 2,2-diphenyl-1-picrylhydrazyl (DPPH^•^) solution in methanol (0.07 mg/mL) were mixed with 20 μL of the extract diluted to various concentrations in 96-well microplates. The solutions were shaken and incubated at 28 °C for 30 min in the dark. A decrease in DPPH^•^ absorbance induced by the sample was measured at 517 nm. A dose response curve for at least five prepared dilutions of each extract was plotted to determine the EC_50_ values. All tests were carried out in triplicate and averaged. The results were expressed as standard equivalents using Trolox (TE) and ascorbic acid (AscE) based on their EC_50_ values. 

### 3.13. ABTS^•+^ Assay

The antiradical activity was assayed using a modified ABTS^•+^ decolorization assay [34]. 20 µL of the extract at different concentrations were mixed with 180 µL of a freshly prepared ABTS^•+^ solution (0.096 mg/mL) in methanol. The mixture was shaken and incubated for 6 minutes at 30 °C in a dark place. The absorbance was measured at 734 nm. The results were expressed as milli-moles of Trolox equivalent per gram of the extract (TEAC). 

### 3.14. Statistical Analysis

All results were expressed as mean ± standard deviation (SD) from three replications. Calculations were performed in STATISTICA 10.0 (StatSoft Poland, Cracow, Poland). The one-way ANOVA test followed by Tukey’s post-hoc test was used for statistical analysis of the differences among the obtained data. Significance was accepted at *p* < 0.05.

## 4. Conclusions

This is the first report revealing the composition and biological activity of crude polysaccharides obtained from *R. rugosa* leaves, achenes, and hips. It broadens the knowledge about the biological potential of CPL from rugosa rose petals as well. The examined polysaccharide-rich fractions demonstrated considerable antioxidant and hyaluronidase-inhibiting potential. Moreover, some of them also exhibited cytotoxic and anti-inflammatory activity. Therefore, it has been shown that crude rose polysaccharides may influence several mechanisms involved in development of some chronic and civilization-related diseases. They affect different biochemical targets related to the chemopreventive potential. Rose hips (pseudofruits) were found to be an abundant and particularly promising source of polysaccharides. CPL-H was characterized by high homogeneity (more than 80% of sugars) and high yield. Due to the high availability of rose raw materials and a relatively easy procedure of CPL preparation, rose crude polysaccharides (especially from petals, leaves and hips) can be considered as an interesting addition to diet, food products, food supplements, or herbal medicines. 

The pre-treatment, type of extraction, and subsequent stages of preparation of CPL were targeted at extraction of polysaccharides from the plant material. However, CPL was found to contain also other groups of active compounds. There is nothing surprising in this fact, since little-processed plant extracts constitute very complex matrices. At this point, it is difficult to establish exactly which components of CPL influence its activity. We are also aware that the demonstrated activity of crude polysaccharides may result from the presence of a mixture of metabolites. The observed biological potential may be connected with synergistic activity of compounds present in an unpurified extract. Further research aimed at detailed investigation of the CPL composition and indication of compounds responsible for the observed activity seems to be an interesting issue, which we have already begun to explore. Moreover, the protein-polysaccharide conjugates found in CLPs are currently being subjected to detailed analysis and the results will be described in the next study.

## Figures and Tables

**Table 1:** Content of water-soluble sugars, total glucans, α - and β -glucans in *R. rugosa* organs (g/100 g of dry weight) and sugar, protein in polysaccharide-rich fractions (% of crude polysaccharide) and total phenolic content (gallic acid equivalents (GaE) per g of CPL.

Plant Part	Water soluble Sugars (g/100 g d.w.)	Total Glucans (g/100 g d.w.)	α-Glucans (g/100 g d.w.)	β-Glucans (g/100 g d.w.)
Petals	0.80 ± 0.00 ^c^	6.67 ± 0.12 ^b^	3.91 ± 0.07 ^c,^*	2.76 ± 0.05 ^c^
Leaves	0.42 ± 0.00 ^b^	2.85 ± 0.04 ^a^	0.94 ± 0.01 ^b^	1.91 ± 0.03 ^b^
Hips	15.37 ± 0.02 ^d^	12.26 ± 0.30 ^c^	5.92 ± 0.11 ^d^	6.35 ± 0.19 ^d^
Achenes	0.21 ± 0.00 ^a^	2.21 ± 0.02 ^a^	0.70 ± 0.00 ^a^	1.52 ± 0.02 ^a^

Values are presented in mean ± standard deviation (*n* = 3) and evaluated by one-way ANOVA test (post test: Tukey). Different superscript letters (^a–d^) in the same column denotes significant differences at *p* < 0.001 (* *p* < 0.05).

**Table 2:** The yield of *R. rugosa* crude polysaccharides (g/100 g of dry weight) and their composition. Sugar and protein content is given in % of crude polysaccharide. Total phenolic content expressed as gallic acid equivalents per g of CPL (GaE). Abbreviations: CPL-Flos—crude polysaccharide from flowers; CPL-Lv—crude polysaccharide from leaves; CPL-H—crude polysaccharide from hips (pseudofruit); CPL-Fru—crude polysaccharide from true fruits (achenes).

Sample	Yield (g/100 g d.w.)	Sugar Content (% of CPL)	Protein Content (% of CPL)	Phenolic Content (GaE)
CPL-Flos	3.81 ± 0.12 ^b^	20.90 ± 0.00 ^b^	0.87 ± 0.03 ^c^	0.22 ± 0.001 ^c^
CPL-Lv	3.20 ± 0.11 ^b^	13.10 ± 0.01 ^a^	0.49 ± 0.01 ^b^	0.11 ± 0.00 ^b^
CPL-H	19.10 ± 0.24 ^c^	80.49 ± 0.11 ^d^	0.23 ± 0.00 ^a^	0.11 ± 0.00 ^b^
CPL-Fru	0.81 ± 0.03 ^a^	25.40 ± 0.02 ^c^	1.08 ± 0.04 ^d^	0.02 ± 0.00 ^a^

Values are presented in mean ± standard deviation (*n* = 3) and evaluated by one-way ANOVA test (post test: Tukey). Different superscript letters (^a–d^) in the same column denotes significant differences at *p* < 0.001.

**Table 3:** Inhibition of COX-1 and COX-2 activity in the presence of *R. rugosa* crude polysaccharides. Abbreviations: n.a.—no activity observed, other abbreviations like in Table 2.

Sample	Extract Concentration (mg/mL)	COX-1 Inhibition (%) Average ± SD	COX-2 Inhibition (%) Average ± SD
CPL-Flos	0.25	14.78 ± 0.21	35.12 ± 1.25
CPL-Flos	0.5	35.21 ± 0.89	49.67 ± 2.42
CPL-Flos	0.75	54.36 ± 1.87	48.71 ± 0.40
CPL-Flos	1	78.42 ± 2.22	67.38 ± 4.81
CPL-Flos	2	92.13 ± 1.64	77.50 ± 1.66
CPL-Flos	5	93.5 ± 2.04	82.06 ± 3.68
CPL-Lv	0.25	5.05 ± 0.13	55.31 ± 2.03
CPL-Lv	0.5	15.12 ± 0.86	68.24 ± 2.15
CPL-Lv	0.75	19.05 ± 0.39	71.86 ± 0.45
CPL-Lv	1	27.34 ± 1.15	79.58 ± 0.53
CPL-Lv	2	33.02 ± 1.44	76.43 ± 3.15
CPL-Lv	5	38.6 ± 1.60	81.10 ± 3.07
CPL-H	0.25	5.26 ± 0.27	6.08 ± 0.12
CPL-H	0.5	12.79 ± 0.84	11.76 ± 0.43
CPL-H	0.75	22.67 ± 0.89	13.46 ± 0.61
CPL-H	1	38.32 ± 1.31	18.88 ± 1.01
CPL-H	2	66.46 ± 1.63	45.0 ± 2.16
CPL-H	5	100 ± 0.0	94.34 ± 2.54
CPL-Fru	0.5	n.a.	n.a.
CPL-Fru	0.75	n.a.	n.a.
CPL-Fru	1	n.a.	n.a.
CPL-Fru	2	n.a.	n.a.
CPL-Fru	5	n.a.	n.a.
Acetylsalicylic acid	1 mM	40.55 ± 1.43	98.21 ± 3.12

**Table 4:** Anti-hyaluronidase potential of *R. rugosa* CPL. Results expressed as mean IC_50_ values ± SD (*n* = 3). Abbreviations like in Table 2.

**Sample**	**IC_50_ (mg/mL)**
CPL-Flos	0.40 ± 0.01 ^a^
CPL-Lv	0.45 ± 0.02 ^a^
CPL-H	6.50 ± 0.14 ^c^
CPL-Fru	1.14 ± 0.01 ^b^
**Standard**	**IC_50_ (mg/mL)**
Methyl indole-3-carboxylate	0.49 ± 0.00
Escin	8.14 ± 0.09
Naringenin	1.28 ± 0.03

Values evaluated by one-way ANOVA test (post test: Tukey). Different superscript letters (^a–c^) in the same column denotes significant differences at *p* < 0.001.

**Table 5:** Antiradical activity of crude polysaccharides. EC_50_ expressed as mg of dry extract per mg DPPH^•^, TE—Trolox equivalent; AscE—ascorbic acid equivalent. Equivalents were calculated by dividing extract EC_50_ by standard EC_50_. For Trolox EC_50_ = 0.09 mg mg^−1^ DPPH^•^; for ascorbic acid EC_50_ = 0.11 mg mg^−1^ DPPH^•^. TEAC—Trolox equivalent antioxidant capacity (assayed with ABTS^•+^ radical cation). Abbreviations: Flos—flower; Lv—leaf; H—hip (pseudofruit); Fru—achenes (true fruit).

Sample	DPPH Scavenging Activity			TEAC
	EC_50_ (mg of Dry Extract /mg DPPH^•^)	TE	AscE	(mmol Trolox/g Dry Extract)
CPL-Flos	0.25 ± 0.01 ^a^	2.86	2.41	1.53 ± 0.03 ^d^
CPL-Lv	0.24 ± 0.01 ^a^	2.70	2.27	0.69 ± 0.02 ^c,^*
CPL-H	1.14 ± 0.02 ^b^	12.76	10.73	0.56 ± 0.03 ^b,^*
CPL-Fru	6.88 ± 0.13 ^c^	76.54	64.38	0.14 ± 0.00 ^a,^*

Values are presented in mean ± standard deviation (*n* = 3) and evaluated by one-way ANOVA test (post test: Tukey). Different superscript letters (^a–d^) in the same column denotes significant differences at *p* < 0.001(* *p* < 0.05).
